# An improved microfluidic device to enhance the enrichment factors in liquid phase microextraction: application to the simultaneous extraction of polar and non-polar acids in biological samples

**DOI:** 10.1007/s00604-023-05752-9

**Published:** 2023-04-04

**Authors:** Alejandro Martín, Rut Fernández-Torres, Miguel Ángel Bello-López, María Ramos-Payán

**Affiliations:** Department of Analytical Chemistry, Faculty of Chemistry, University of Seville, C/Prof., García González S/N, 41012 Seville, Spain

**Keywords:** Microfluidic, Liquid phase microextraction, Supported liquid membrane, Sample treatment, Acidic drugs

## Abstract

A new microfluidic device to enhance the enrichment factor in miniaturized systems is proposed. The microfluidic system was design for liquid phase microextractions, and it was applied to the simultaneous extraction of acidic compounds of a wide range of polarity (0.5 < log *P* < 3). The device operated under stagnant acceptor phase conditions and all the operational parameters involved were optimized. Tributyl phosphate was found to be a new highly efficient supported liquid membrane to simultaneously extract analytes of very different polarities. The optimal donor and acceptor phase were pH 2 and pH 13, respectively. The donor flow rate and the extraction time were investigated simultaneously, offering great versatility with high enrichment factors (EFs). Limits of quantitation were within 0.02 and 0.09 µg mL^−1^ for all compounds at 10 µL min^−1^ as donor flow rate and 20-min extractions, offering EFs between 11 and 18 with only 200-µL sample volume consumption. The method was successfully applied to human urine samples, observing recoveries between 47 and 90% for all compounds. This new proposed microfluidic system increases the wide range of applications, especially when the analytes are present in lower concentrations in the sample.

**Graphical Abstract:**

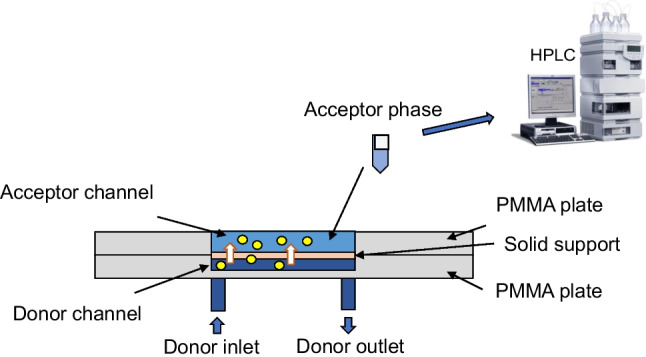

## Introduction

Microfluidic devices have undergone significant improvements over the past decade, with advances in fabrication techniques, materials, integration, and applications [1]. These improvements have enabled the development of more advanced and sophisticated microfluidic devices, which are being used in a wide range of applications, including drug delivery [2], diagnostics [3], environmental monitoring [4, 5], and biological sample analysis [6–9]. The field of microfluidics has been extensively developed in sample treatment during the last decades towards improvement in efficiency, reduction of sample volume, use of hazardous solvents, extraction time, and better integration and compatibility with different detection methods [10–12]. Liquid phase microextraction [13, 14] and electromembrane extraction [15, 16] are two successfully implemented techniques in microfluidic systems [17–19]. The downscaling of these techniques is becoming increasingly popular due to their advantages of low cost, low sample consumption, and high selectivity compared to traditional setups. In addition, these devices can be fabricated using relatively inexpensive materials (such as polymethylmethacrylate) and processes, making them cost-effective compared to other techniques or devices. These systems were initially reported for the extraction of different families of compounds [6, 20–23] by LPME or the individual extraction of compounds with similar acidic or basic properties by EME [24–28]. These devices have also been developed to challenge the simultaneous extraction of compounds of different families, chemical properties, or polarities. These advances were achieved not only due to the investigation of new supported liquid membranes [29–31] but also with new geometric proposals. For example, LPME and EME were integrated on one chip for the simultaneous extraction of different families [32]. Later, two new geometries were reported for the simultaneous extraction of acidic and basic compounds by double EME/EME [33, 34]. One key aspect of microfluidic systems is the geometry of the fluidic channels, which can have a significant impact on their performance. For example, one of the advantages of working under low flow conditions is the low sample consumption required, which increases in semi-continuous systems (stagnant acceptor phase). Most microfluidic systems have been reported to improve extraction efficiencies under double flow rate [6, 21, 28, 32, 34, 36] with no enrichment factor. Very few LPME and EME-based microfluidic systems have shown some enrichment factor under stagnant conditions [20, 24, 25, 27], this being one of the great limitations and drawbacks of these systems in LPME and EME. The need to improve the geometry of microfluidic systems to enhance the enrichment factor is driven by the desire to make these systems more effective and efficient for a wide range of applications, in particular where the analyte has a low concentration in the sample.

More research into this area is needed to address some existing challenges and make more improvements in the reported geometries. In this work, we investigate new microfluidic geometries and supported liquid membranes to enhance the enrichment factor for the simultaneous extraction of acidic compounds of a wide range of polarity (0.5 < log *P* > 3).

## Experimental

### Chemicals and sample solutions

All reagents and chemicals were of analytical grade. Hippuric acid (HIP), anthranilic acid (ANT), ketoprofen (KET), naproxen (NAP), 1-octanol, 1-decanol, formic acid, methanol, and chloride acid were purchased from Fluka–Sigma–Aldrich (Madrid, Spain). Sodium hydroxide, 2-nitrophenyl octyl ether (NPOE), nonanol, decanol, undecanol, and tributyl phosphate (TBP) were supplied from Merck (Darmstadt, Germany). The stock solutions of polar and non-polar acidic compounds were prepared in methanol at 200 mg L^−1^ and preserved at 4 °C. Working solutions were daily prepared from stock solutions by adequate dilutions with deionized water (Milli-Q Plus water purification System). A flat membrane (Celgard 2500 with 25 μm thickness, 55% porosity, and 0.21 μm × 0.05 μm pores) was used as solid support for the supported liquid membrane, and one micro-syringe pump (Cetoni GmbH, Korbussen, Germany) was used to introduce the donor phase into the microfluidic device.

### Fabrication and setup of the microfluidic device

**Fig. 1:**
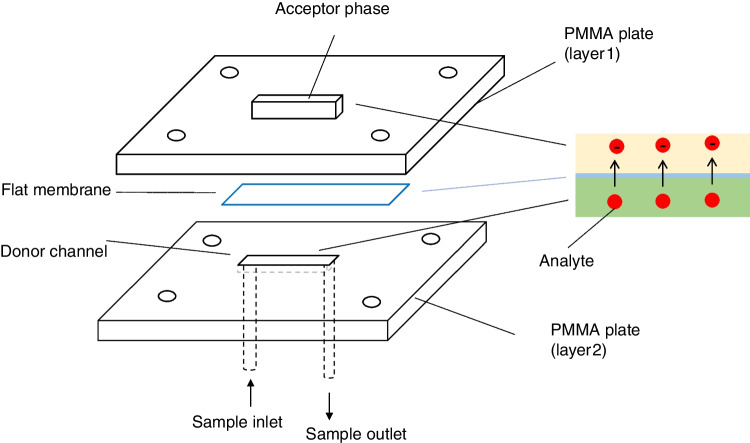
Scheme of the microfluidic device based liquid-phase microextraction

A scheme of the new microfluidic device is shown in Fig. 1. The device was made of poly(methyl methacrylate) (PMMA) and it contained two compartments. The bottom layer (layer 1) contains a channel of 10 mm length, 2 mm width, and 0.12 mm depth. The top layer (layer 2) contains a hollow of 6 mm length, 2 mm width, and 2 mm depth. The device was fabricated using an Epilog Mini 24–30 W laser cutter and the conditions of ablation for the donor channel were 35% for writing speed and power, a resolution of 1500, and a frequency of 5000. For the acceptor channel, 10 and 90% for writing speed and power were used. The bottom channel and the top channel were designed to locate the donor and the acceptor solutions, respectively. The inlet and outlet connections of the donor (sample) solution were made by drilling two holes at the beginning and the end of the donor channel. Teflon tubes of 1.5 mm were used to introduce the donor solution into the channel. The layer 1 was located on the top of the donor PMMA plate (layer 2). A flat membrane (Celgard 2500) was located between both channels to separate the donor and the acceptor phases, and it was previously impregnated with 3 µL of TBP to form the supported liquid membrane. Finally, the device was assembled by using 4 screws (3 mm o.d.) and the donor solution (containing 1 mg L^−1^ of each analyte) was connected to the micro-syringe pump (Cetoni GmbH, Korbussen, Germany) to correctly adjust the flow rate. The amount of sample introduced into the syringe will depend on the amount of sample required for the stabilization of the device and the extractions to be carried out. In this case, 700 µL of donor phase was appropriate to carry out several consecutive extractions. The device is versatile and can operate using different flows and extraction times. In this work, a flow of 10 µL min^−1^ and 20 min of extraction were selected to demonstrate its applicability in real samples. The acceptor solution was collected after 12 min of extraction and it was analyzed by HPLC.

### Chromatographic conditions

A VWR-Hitachi (Barcelona, Spain) liquid chromatograph with a quaternary L-7100 pump, a L-7455 diode array detector, and an autosampler L-2200 as injector was used for the analysis of the analytes. A LiChroCART 75–4 Purosphere STAR RP-18e 3 µm (75 mm × 4.0 mm i.d.) (VWR, Germany) proceeded by a guard column Kromasil1 100 Å, C18, 5 µm (20 mm × 4.6 mm i.d.) (Scharlab S.L., Barcelona, Spain) was used for the separation at room temperature. Formic acid 0.1% (component A) and methanol (component B) were used as mobile phases, and 0.6 mL min^−1^ was set as the flow rate. The chromatographic separation was performed using the following gradient: 30% B from 0 to 1 min and from 30 to 100% B for 11 min. Three minutes was set for column re-equilibration between injections to the initial conditions. The wavelengths for HIP, ANT, KET, and NAP were 235, 235, 255, and 230 nm, respectively. The separation was completed in 10 min.

### Preparation of real samples

Urine samples from a 30-year-old male volunteers (prior consent) were collected and adjusted to pH 13. Non-diluted and diluted samples (1:1) were spiked at 0.1, 0.6, and 1.5 mg L^−1^ and all samples were filtered through Pall NylafloTM nylon membrane filter 0.45 µm (Pall Corporation, Ann Arbor, Michigan, USA).

### Calculations of extraction efficiency and enrichment factor

The enrichment factor (*EF*_*i*_) for the analyte *i* was calculated according to the following Eq. (1):

1 $${EF}_{i}=\frac{{C}_{f,a,outlet}}{{C}_{i,s,inlet}}$$

where ${C}_{f,a,outlet}$ is the concentration of the analyte *i* at the outlet of the acceptor channel and ${C}_{i,s,inlet}$ is the initial concentration of the analyte in the sample.

The extraction efficiency (*EE*) was defined as the fraction of analyte transferred to the acceptor phase from the sample. Using a double-flow working mode, the extraction efficiency (*EE*%) was calculated according to the following Eq. (2):

2 $$EE\;\left(\%\right)={EF}_i\;x\frac{v_a}{v_s}x\;100$$

where $v_a\;\mathrm{and}\;v_s$ are the acceptor and sample volumes, respectively.

## Results and discussion

### Supported liquid membrane selection

The selection of the supported liquid membrane is one of the critical parameters in liquid-phase microextraction procedures using a membrane as a solid support. Based on previously reported works, different organic solvents have been tested for the extraction of non-polar acidic compounds [7] and simultaneous extraction of polar and non-polar acidic compounds [35]. Although decanol and NPOE have shown to be good SLMs in previously published studies for the extraction of acidic compounds, it is important to investigate new SLMs as alternatives to improve efficiencies or enrichments, especially in newer semi-static devices. Then, octanol, DHE, undecanol, nonanol, decanol, NPOE, and TBP were tested as SLM. For these preliminary studies, the pH of the donor (containing 1 mg L^−1^ of all compounds) and the acceptor phase were set at pH 12 and pH 3, respectively, based on the basic principles of the liquid-phase microextraction by passive diffusion, where the analytes must be neutral in the donor phase and charged in the acceptor phase. Since their pK_a_ range is between 4 and 6 for all the analytes, then the acidic compounds were neutral and negatively charged in the donor phase and acceptor phase, respectively. The donor flow rate was set at 20 µL min^−1^ and the extraction time was fixed at 15 min, to increase the enrichment factor, since our previous studies [7] showed that an increase of the donor flow rate increased the enrichment under double-flow conditions. The volume of the stationary phase was initially set at 15 µL. Table 1 summarizes the efficiencies and enrichment obtained for each organic solvent tested. Polar compounds were not extracted with DHE and NPOE showed better extractions for non-polar compounds. In the same way, alcohols with different chain carbon numbers showed better extractions for non-polar compounds. As seen in Table 1, TBP showed the highest enrichment for all compounds within a log P range between 0.5 and 3. Furthermore, higher extraction efficiencies were obtained with TBP (between 18 and 55%). Each experimental point was carried out in triplicate, observing a relative standard deviation below 3% for all compounds. Compared to previously microfluidic methods for the extraction of acidic compounds, this new SLM (TBP) showed a better response for the simultaneous extraction of polar and non-polar acidic compounds using a new microfluidic device which works under semi-continuous conditions. Then, TBP was selected as a supported liquid membrane for the rest of the study.

**Table 1:** Extraction efficiencies (RSD %) of polar and non-polar acidic compounds using different organic solvents as SLM

SLM	Extraction efficiency ± SD (%)			
	HIP	ANT	KET	NAP
NPOE	5.3 ± 0.2	8.8 ± 0.4	33.1 ± 1.8	46.5 ± 1.6
Undecanol	21.7 ± 0.2	14.7 ± 1.9	38.4 ± 1.6	39.7 ± 2.1
Decanol	13.2 ± 1.2	10.3 ± 0.7	27.6 ± 1.1	31.2 ± 2.0
TBP	16.3 ± 0.8	55.1 ± 1.8	45.2 ± 0.9	44.8 ± 0.9
Octanol	28.0 ± 1.4	10.0 ± 0.8	42.6 ± 2.1	43.0 ± 1.5
DHE	*	*	44.7 ± 1.8	39.1 ± 1.6
Nonanol	0.60 ± 0.03	17.0 ± 0.3	36.6 ± 1.3	33.7 ± 1.7

^*^Non-detected

### Optimization of the donor and the acceptor phase composition

**Fig. 2:**
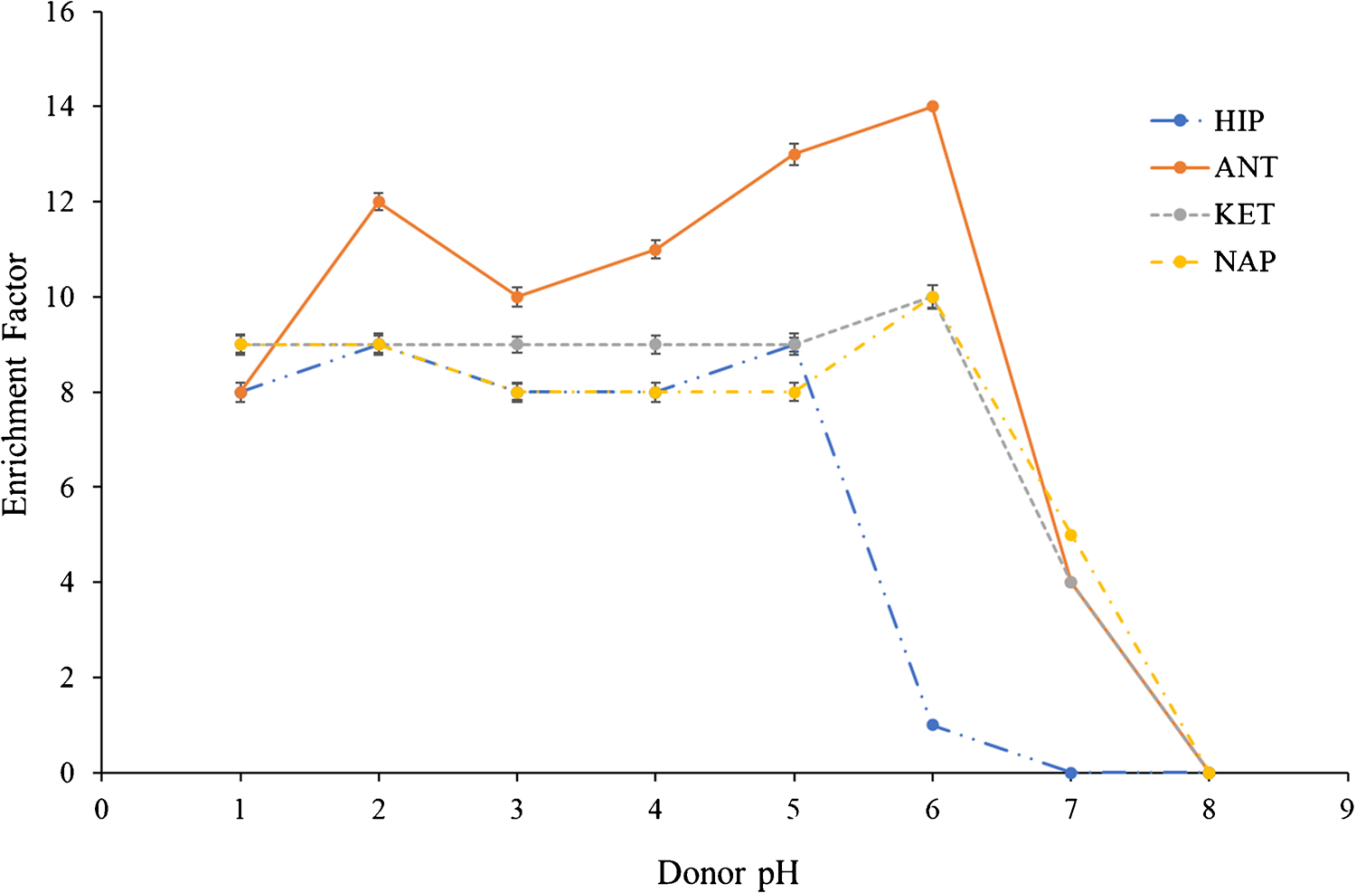
Optimization of the donor phase composition. Experimental conditions: TBP (as SLM), pH 12 (acceptor phase composition), 20 μL min.^−1^ (donor flow rate), and 15 min (extraction time)

The compositions of the donor phase and the acceptor phase are decisive for passive diffusion, which occurs thanks to a pH gradient between both phases. TBP was set as the new SLM to study the trend of the composition of both phases. For the experiments, the donor flow rate and the extraction time were set at 20 µL min^−1^ and 15 min, respectively. First, the donor phase composition was studied between a range of 1 and 8 using a pH 12 as acceptor phase composition. As seen in Fig. 2, a similar behavior was observed for non-polar acidic compounds across the pH range studied. However, the polar compounds behaved slightly differently, observing a significant decrease in the EF for the HIP and the ANT at pH 5 and 6, respectively. A clear decrease in the enrichment factor was observed for all the compounds close to their pK_a_, expected according to the phenomenon of diffusion in the liquid phase. However, no significant differences were observed between pH 2 and pH 5, except for ANT. Then, a pH 2 (HCl) was set as the optimal donor pH for all compounds. Each experimental point was tested in triplicate and RSD below 3% was observed for all experiments.

**Fig. 3:**
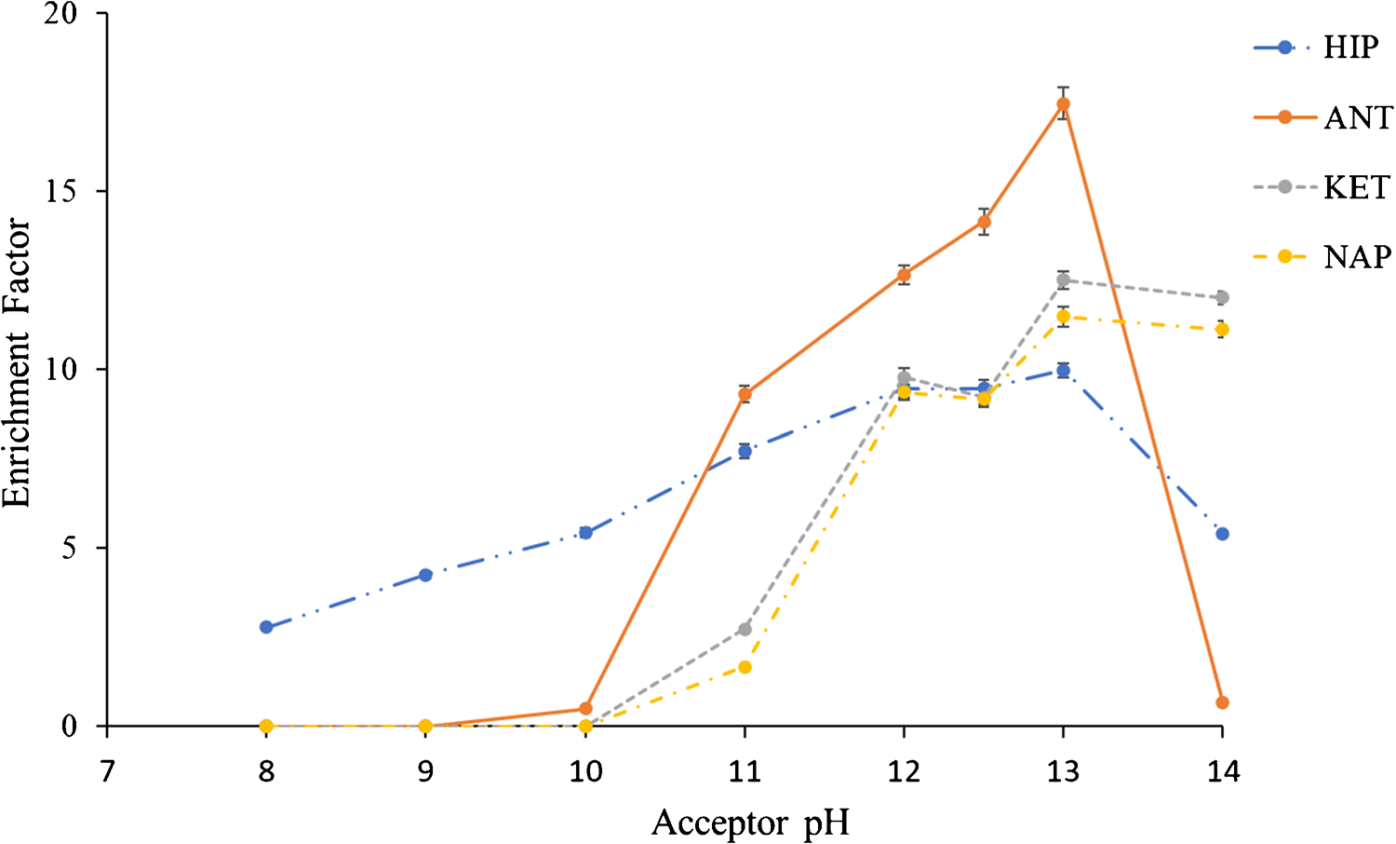
Optimization of the acceptor phase composition. Experimental conditions: TBP (as SLM), pH 2 (donor phase), 20 μL min.^−1^ (donor flow rate), 15 min (extraction time)

The acceptor pH composition was studied between pH 8 and 14. As seen in Fig. 3, an increase in the enrichment factor was observed up to pH 13 for polar and non-polar compounds, so pH 13 was set as the optimal acceptor phase. A possible degradation of ANT at high pH may be related to the significant decrease at pH above 13. Each experimental point was tested in triplicate and relative standard deviations below 2% were obtained for all cases. The pH stability was checked before and after extraction and no significant changes were observed. The membrane was replaced after each experiment and a new SLM was added to test each different SLM. The highest extraction efficiencies (between 45 and 65% for all compounds) were also obtained at pH 2. The trends of the composition of the donor and acceptor phases (Figs. 2 and 3) are different from those previously studied using different devices under double flow [7, 35]. These results suggest the need to optimize each of the operational parameters if both the SLM and the geometry of the device change.

### Donor flow rate and extraction time optimization

**Fig. 4:**
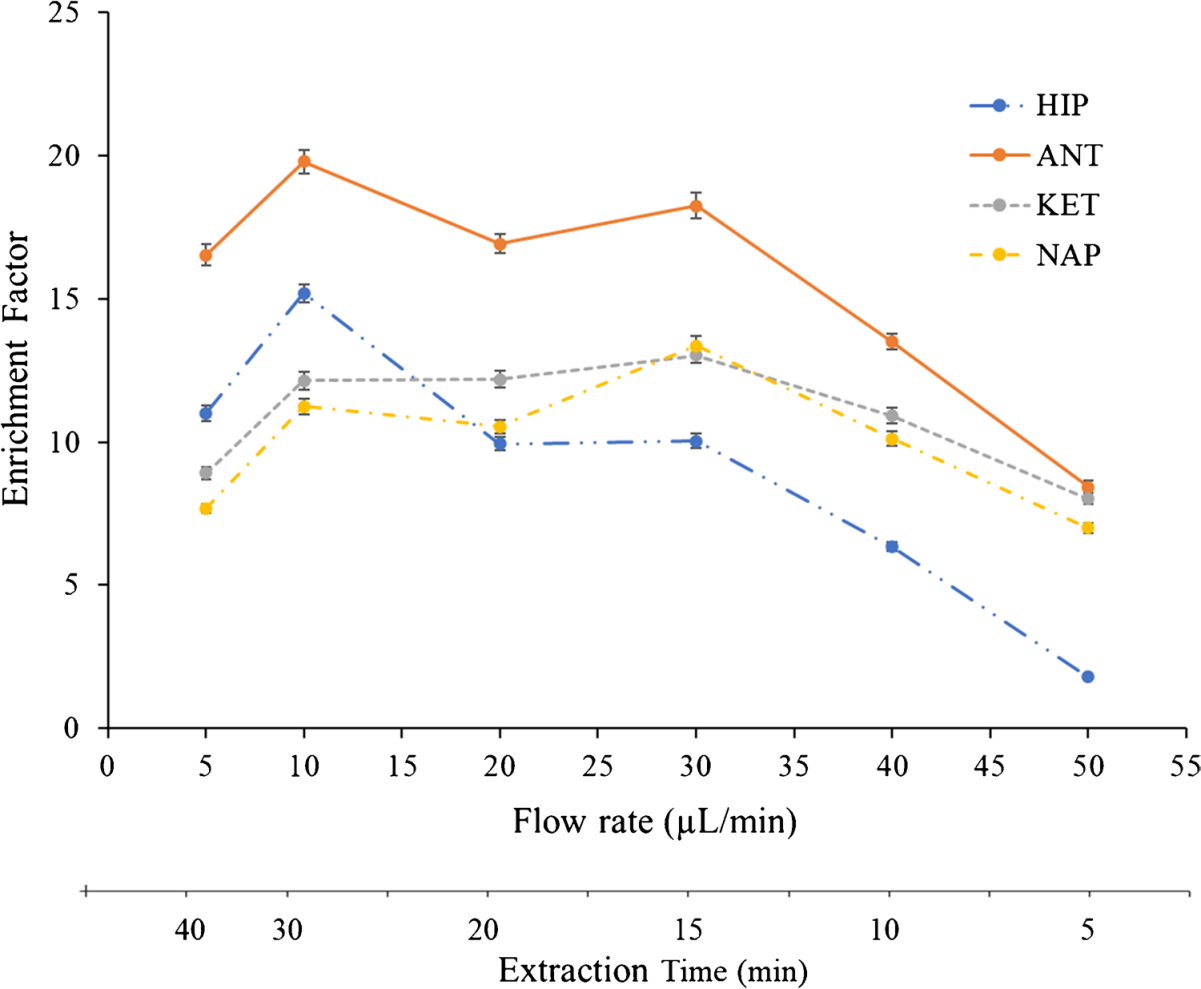
Study of the donor flow rate and the extraction time: enrichment factor versus donor flow rate. Experimental conditions: TBP (as SLM), pH 2 (donor phase), pH 13 (acceptor phase)

The donor flow rate was optimized under semi-continuous flow conditions in microfluidic systems. Unlike double-flow devices, the acceptor phase flow is stationary, and the donor flow should be optimized together with the extraction time. Firstly, an optimization was carried out combining different flows and different times as shown in Fig. 4. Enrichments between 11 and 20 were observed for all compounds at a flow rate of 10 µL min^−1^ and 30 min of extraction, with a sample consumption of 300 µL. As expected, the extraction efficiencies decreased as the donor flow increased due to a slow passive diffusion, while a lower flow rate and longer extraction times offered higher extraction efficiencies.

In parallel, five different donor flow rates (1, 5, 10, 20, and 30 µL min^−1^) were individually tested at different extraction times (5, 10, 15, 20, and 30 min). A flow rate greater than 40 µL min^−1^ was not tested since a significant increase in the standard deviation (*RSD* > 15%) and an instability of the laminar regime of the donor phase were observed. Table 2 shows the different flow rates between 5 and 30 min. As seen in Table 2, a flow rate of 1 µL min^−1^ did not show an enrichment factor over 2 and the EF increased when increasing the donor phase flow rate and extraction time for all compounds. Low flows and longer extraction times showed similar EFs compared to extractions at higher flows and shorter extraction times, the latter being the most desirable option to achieve faster extractions. For example, enrichment factors between 10–16 and 10–15 were obtained at 20/20 (flow rate/extraction time) and 30/15 (flow rate/extraction time), respectively. The sample volume consumption was 50 µL lower for 20/20 compared to 30/15 combination; however, the extraction time was 5 min longer. Different combinations between the donor flow rate and the extraction time could be selected based on the enrichments required for the analysis in real samples and the sample volume availability. Membrane was replaced between each different experiment and its stability for carrying out consecutive extraction was also studied under the same experimental conditions. The results showed that three consecutive extractions could be carried out, observing an RSD below 3% for all compounds and a good reusability of the SLM for more than one extraction. Those experiments were tested with and without replacing the SLM between extractions, observing the same results for both cases. The proposed microfluidic system provides the versatility to work at different times and flows based on the need for pre-concentration in the sample. High EFs between 11 and 18 (10 µL min^−1^ as a flow rate and 20 min) using only 200 µL of sample were obtained.

**Table 2:** Enrichment factors at different donor flow rates and extraction times for all compounds

Enrichment factor						
		Extraction time (min)				
Analyte	Flow rate (µL/min)	5	10	15	20	30
HIP	1	0.93	0.98	0.98	1.03	1.14
	5	1.61	2.27	2.98	3.68	7.78
	10	1.86	4.20	6.19	10.85	15.00
	20	1.97	3.76	6.97	9.94	20.10
	30	2.33	4.15	10.03	14.93	23.18
ANT	1	0.61	0.68	1.28	1.63	1.84
	5	1.17	3.37	4.36	5.35	11.35
	10	2.33	7.19	11.76	17.89	19.79
	20	3.11	8.85	12.88	16.92	28.26
	30	5.76	10.58	15.76	19.88	32.77
KET	1	0.73	0.88	0.85	0.91	1.03
	5	1.11	1.21	1.88	2.58	7.27
	10	3.62	5.00	8.30	12.60	12.1
	20	4.77	7.95	10.10	12.18	22.33
	30	5.83	8.37	13.02	14.33	24.74
NAP	1	0.70	0.85	0.90	0.93	1.04
	5	0.97	1.11	1.76	2.27	5.68
	10	1.15	4.06	6.33	9.71	11.24
	20	3.26	6.21	9.15	10.54	18.26
	30	4.45	7.48	13.30	17.85	22.40

The miniaturization of sample treatment systems implementing liquid-phase microextraction or electromembrane extraction still presents some limitations, especially with respect to the enrichment factor. In the last decade, the design of these devices has evolved to make these devices reusable. In most cases, these LPME-based devices have shown great extraction efficiency under double-flow conditions, allowing consecutive extractions [17]. There are also EME-based devices that are reusable and that were designed to work under stopped flow conditions, offering enrichments between 13 and 16 [24, 25] using extraction times greater than 30 min and 1 mL of sample consumption. However, low or inexistent enrichment factors remain a limitation and challenge in microfluidics. In general, these designs fundamentally differ in the length and depth of their channels. For example, shallower channels are used for LPME under double-flow conditions, while deeper channels have been used in EME since additional electrode placement is needed in the channel. Furthermore, in previously proposed designs, the device required its opening to regenerate the SLM. The new version of the proposed design consists of improving the versatility of these devices compared to those previously described, improving (or equalling) the enrichment factors but decreasing the required sample volume and extraction times. Additionally, this design allows the SLM to be filled without the need to open the device, thus saving time and improving reproducibility. Although there are hollow fiber-based LPME methods that offer higher enrichments [36], they require a much larger sample volume and longer extraction times, and hence, the importance of investigating new miniaturized designs. This new microfluidic method simultaneously extracts polar and non-polar acidic compounds offering good enrichment factors (11–18), it reduces the sample volume consumption 4 times compared to other stagnant microfluidic methods, it reduces the extraction time, and it is low-cost and simple to handle since it does not need a power supply. Higher enrichment factors (up to 32) can be achieved by consuming 900 µL of sample and completing the extraction in 30 min.

## Evaluation of analytical performance

The microfluidic device was evaluated for the simultaneous extraction of polar and non-polar acidic compounds in aqueous solutions, selecting a pH 2 as donor phase, pH 13 as acceptor phase, TBP as SLM, 10 µL min^−1^ as a flow rate, and 20 min of extraction. The analytical validation was based on international guidelines [37]. A calibration curve was obtained using a least-square linear regression analysis from 0.05 to 5 mg L^−1^ for HIP, from 0.02 to 5 mg L^−1^ for ANT, from 0.03 to 5 mg L^−1^ for KET, and from 0.09 to 5 mg L^−1^ for NAP. A linear relationship with *r*^2^ values over 0.9989 was observed for all analytes. Table 3 shows the detection limits (based on S/N ratio of 3), quantitation limits (based on S/N ratio of 10), linear range, regression coefficients, and the enrichment factors for each analyte. Inter-day precision and intra-day precision were studied in triplicate (*n* = 3) at low, medium, and high levels of the calibration curve for each compound, observing an RSD (%) between 1 and 3% for inter-day precision and an RSD (%) between 2 and 3% for intraday precision, using different membranes in both cases. Under the operational parameter conditions described above, the enrichment factors were 11, 18, 13, and 18 for HIP, ANT, KET, and NAP, respectively.

**Table 3:** Calibration parameters, detection limit (LOD), quantitation limit (LOQ), and enrichment factors at 10 μL min^−1^ and 20 min extraction

	LOD^a^ (µg mL^−1^)	LOQ^a^ (µg mL^−1^)	*R*^2a^	Linear range^a^ (µg mL^−1^)	EF^*,a^
HIP	0.015	0.05	0.9989	0.05–5	11
ANT	0.006	0.02	0.9992	0.02–5	18
KET	0.009	0.03	0.9991	0.03–5	13
NAP	0.027	0.09	0.9991	0.09–5	18

^*^Enrichment factor (%RSD, *n* = 3)

^a^Extraction at 10 µL min^−1^ donor flow rate and 20-min extraction. Sample volume consumption: 200 µL

## Urine sample analysis

The microfluidic device was tested on human urine samples. Human urine samples were collected from a healthy adult male volunteer. Non-diluted and diluted samples (1:1) with Milli-Q water were adjusted to pH 13 and spiked at three different concentrations within the calibration curve range of each analyte. The results are shown in Table 4, observing an increase in the recovery for 1:1 diluted sample. Recoveries were calculated comparing the extraction efficiencies in aqueous solutions with those obtained in human urine samples. As seen in the table, recoveries between 72 and 89% were obtained for all analytes (1:1 diluted samples), except for ANT (44%), and the relative standard deviations were below 3% in all cases. A higher 1:2 urine sample dilution offers recoveries of ANT over 75%; so considering its EF (18) and the expected concentration values in urine, it would be possible to use 1:2 or even higher urine dilutions to improve spiked recoveries of ANT.

**Table 4:** Recoveries (average of three determinations ± standard deviation) from spiked urine samples at different concentrations

Urine samples	Concentration level (µg mL^−1^)	Recovery (%) ± SD (%) (*n* = 3)			
		HIP	ANT	KET	NAP
Non-diluted	0.1 0.6 1.5	74 ± 2	33 ± 1	36 ± 2	34 ± 3
		73 ± 1	34 ± 1	35 ± 1	33 ± 1
		74 ± 1	35 ± 2	36 ± 2	35 ± 2
1:1 dilution	0.1 0.6 1.5	89 ± 3	47 ± 2	75 ± 2	71 ± 3
		88 ± 2	45 ± 3	77 ± 1	78 ± 2
		91 ± 2	44 ± 2	77 ± 2	76 ± 3

**Fig. 5:**
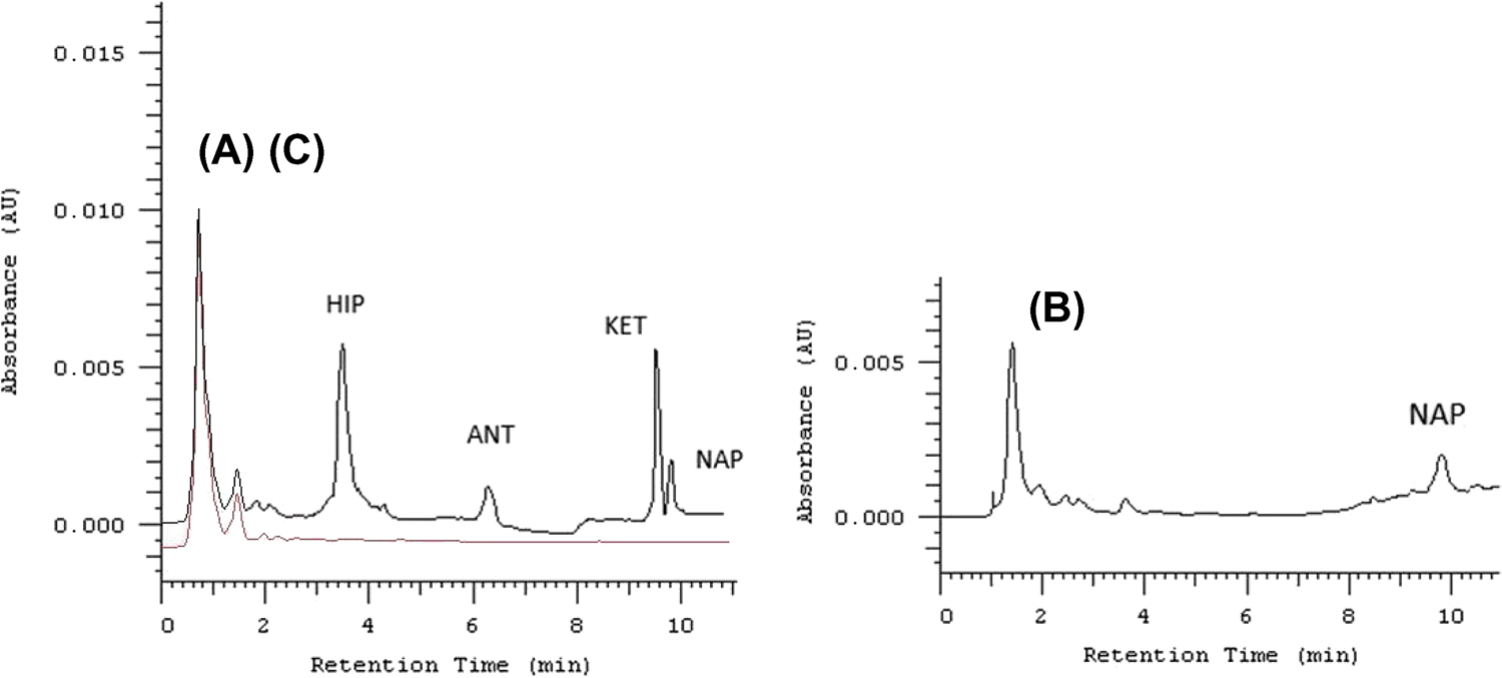
Chromatogram of a **A** spiked urine sample after microfluidic extraction, **B** human urine sample collected 3 h after an oral administration of 550 mg of naproxen, and **C** blank human urine sample

The LOQ values offered of the proposed microfluidic method are much lower compared to the usual values of these compounds in human urine samples (300–500 µg mL^−1^ for HIP, 5 µg mL^−1^ for ANT, 160 µg mL^−1^ for KTP) [38–41]. Human urine collected from a healthy volunteer after the administration of an oral doses of NAP was microfiltered and diluted with ultrapure water (1:1, v/v) and submitted to the microfluidic device for its extraction. According to the literature, 95% of NAP approximately of this drug is excreted in the urine, being 66–92% as conjugates, < 1% as 6–0-desmethyl-naproxen, and < 1% as NAP [42]. Considering the collected volume of urine as well as the enrichment factor and the spiked recovery under selected operational conditions (10 µL min^−1^ donor flow rate and 20 min extraction), the concentration found (0.38 µg mL^−1^) for NAP in human urine samples is in accordance with the usual excreted amounts according to the literature data. These results agree with the usual excreted levels in the period corresponding from the ingestion to the collection (3 h). Therefore, it can be assessed that the proposed microfluidic method could be applied for determining these analytes in real urine samples. Figure 5 shows the chromatograms corresponding to (A) human urine spiked samples containing all analytes, (B) human urine sample collected 3 h after an oral administration of 550 mg of naproxen, and (C) a blank human urine sample, observing an excellent clean-up for 1:1 diluted urine samples under stagnant conditions (acceptor phase).

## Conclusions

For the first time, an improved microfluidic device has been developed to increase the enrichment factor for simultaneous extraction of polar and non-polar acidic compounds in a large log P window (0.5 < log *P* < 3). The continuous development of new miniaturized techniques is of special importance to improve portability, reduce costs, and shorten analysis procedures, but above all, it is important to gradually reduce the limitations they present. This new geometry addresses the limitations regarding the low enrichments offered by these systems today under stagnant conditions, by increasing the enrichment factor (lower LOQs), decreasing the extraction time (20 min), and the sample volume consumption (200 µL) at 10 µL min^−1^ as the flow rate. This versatile device showed good stability and reproducibility at different donor flow rates and extraction times, offering higher EFs (up to 32) by increasing the extraction time and the sample flow rate, if needed. The geometry of the systems can have a significant impact on the performance of microfluidic methods, improving sensitivity and LODs, allowing the analysis of compounds that are present at low concentrations in real samples, such as water samples. The microfluidic device requires the order of microliters of sample, and although the amount of urine sample available is not limited, other types of biological samples in which it could be applied are newborn urine, amniotic fluid, etc., where the amount of sample available is limited.
